# Tolerogenic Properties of Lymphatic Endothelial Cells Are Controlled by the Lymph Node Microenvironment

**DOI:** 10.1371/journal.pone.0087740

**Published:** 2014-02-04

**Authors:** Jarish N. Cohen, Eric F. Tewalt, Sherin J. Rouhani, Erica L. Buonomo, Amber N. Bruce, Xiaojiang Xu, Stefan Bekiranov, Yang-Xin Fu, Victor H. Engelhard

**Affiliations:** 1 Department of Microbiology, Immunology, and Cancer Biology and Carter Immunology Center, University of Virginia School of Medicine, Charlottesville, Virginia, United States of America; 2 Department of Biochemistry and Molecular Genetics, University of Virginia School of Medicine, Charlottesville, Virginia, United States of America; 3 Department of Pathology, University of Chicago, Chicago, Illinois, United States of America; New York University, United States of America

## Abstract

Peripheral self-tolerance eliminates lymphocytes specific for tissue-specific antigens not encountered in the thymus. Recently, we demonstrated that lymphatic endothelial cells in mice directly express peripheral tissue antigens, including tyrosinase, and induce deletion of specific CD8 T cells via Programmed Death Ligand-1 (PD-L1). Here, we demonstrate that high-level expression of peripheral tissue antigens and PD-L1 is confined to lymphatic endothelial cells in lymph nodes, as opposed to tissue (diaphragm and colon) lymphatics. Lymphatic endothelial cells in the lymph node medullary sinus express the highest levels of peripheral tissue antigens and PD-L1, and are the only subpopulation that expresses tyrosinase epitope. The representation of lymphatic endothelial cells in the medullary sinus expressing high-level PD-L1, which is necessary for normal CD8 T cell deletion kinetics, is controlled by lymphotoxin-β receptor signaling and B cells. Lymphatic endothelial cells from neonatal mice do not express high-level PD-L1 or present tyrosinase epitope. This work uncovers a critical role for the lymph node microenvironment in endowing lymphatic endothelial cells with potent tolerogenic properties.

## Introduction

Self-reactive T cells that have escaped negative selection in the thymus are tolerized by both extrinsic and intrinsic mechanisms in the periphery. It is well established that dendritic cells (DC) present self-antigen acquired from dead or dying cells in tissues to self-reactive T cells in draining lymph nodes (LN) and induce anergy [1] or deletion [2]–[4], a process known as cross-tolerance. Recently, three groups, including our own, demonstrated that LN stromal cells (LNSC) directly express peripheral tissue antigens (PTA), genes normally restricted to one or a few tissues, and mediate deletion of self-reactive CD8 T cells [5]–[7]. Four distinct CD45^neg^ LNSC populations can be distinguished based on expression of gp38 and CD31: lymphatic endothelial cells (LEC), blood endothelial cells (BEC), fibroblastic reticular cells (FRC), and double negative (DN) cells [8]. Thus far, LEC, FRC, and extra-thymic cells that express the autoimmune regulatory element (Aire) (eTAC), which are a subset in the DN compartment, have been shown to act in this way [9]–[11].

Tyrosinase, a melanocyte differentiation protein involved in pigment production, encodes an HLA-A*0201-restricted epitope, Tyr_369_, which is associated with autoimmune vitiligo and also a target of melanoma immunotherapy [12], [13]. We showed that mice that transgenically express a chimeric HLA-A*0201-based molecule [14], termed ‘AAD’, do not enforce CD8 T cell peripheral tolerance to Tyr_369_ via negative selection [6]. Instead, tyrosinase-specific CD8 T cells underwent activation and deletion in LN. This peripheral tolerance induction was not mediated by conventional DC or Langerhans cells [6] and instead was due to LN-resident LEC (LN-LEC) that directly express tyrosinase mRNA independent of Aire [10]. LN-LEC also express PTA characteristic of the pancreas and intestine, suggesting that they play a broad role in peripheral tolerance. More recently, we demonstrated that deletion of CD8 T cells bearing a transgenic TCR specific for Tyr_369_, termed ‘FH’, is due to their engagement with PD-L1 expressed by radio-resistant LN cells and that LEC express the highest level of PD-L1 among LNSC subpopulations [15]. Overall, our results support a model of systemic peripheral tolerance in which potentially auto-reactive naïve CD8 T cells enter LN, engage self-antigen and PD-L1 expressed on LN-LEC, and undergo deletion.

LEC also comprise the afferent lymphatic vessels present in most tissues [16], [17], which maintain fluid homeostasis, and facilitate the movement of soluble antigens and hematopoietic cells into LN [17]. Afferent lymphatic LEC express chemokines and adhesion molecules that facilitate the migration of DC and T cells to LN [18]. Although the predominant route of naïve T cell circulation into LN occurs via the blood circulation, a significant number of naïve T cells are also found in afferent lymph [19]. Thus, naïve CD8 T cells have the potential to interact with LEC in afferent lymphatic vessels as well as those in LN. However, it is unknown whether peripheral tissue LEC and LN-LEC have similar tolerogenic potential.

LEC occupy three distinct locations in the LN: the subcapsular sinus, the medullary sinus, and blind-ended sinuses in the cortex. LN-LEC produce CCL19 and CCL21, which attract lymphocytes into the LN [20]–[22] and sphingosine-1-phosphate (S1P), which promotes lymphocyte egress [23], [24]. Lymphocytes interact with subcapsular LEC as they migrate in from afferent lymphatics, with cortical LEC as they enter and exit the lumen of the LN, and with medullary LEC as they exit into the efferent lymphatics. However, phenotypic discrimination of these anatomically distinct LEC subpopulations is lacking and differences in their tolerogenic properties, including expression of PTA and PD-L1, remain unexplored.

In this study, we identified phenotypic characteristics that distinguish LEC in peripheral tissues and those that occupy different areas of the LN. We evaluated their expression of PD-L1 and tyrosinase, and their ability to present Tyr_369_. Finally, we evaluated molecular and cellular pathways that might control these characteristics. Overall, our results demonstrate that the LN microenvironment promotes multiple tolerogenic aspects of LEC.

## Materials and Methods

### Ethics Statement

This study was carried out in strict accordance with the recommendations in the Guide for the Care and Use of Laboratory Animals of the National Institutes of Health. Procedures were approved by the University of Virginia Animal Care and Use Committee (Animal Welfare Assurance # A3245-01). All efforts were made to minimize suffering.

### Mice

C57BL/6 mice carrying the AAD transgene or a fully deleted tyrosinase gene (c^38R145L^) have been described [14], [25]. FH mice were generated using TCR genes from a Tyr_369_ specific T cell clone derived from an AAD^+^ albino mouse [6]. µMT^−/−^, Rag1^−/−^,TNFα^−/−^, and Batf3^−/−^ mice (C57BL/6) and CD11c-DTR mice (Balb/c) were from The Jackson Laboratory. CD11c-DTR mice were treated with diphtheria toxin (DT) s.c. every day for 1 week to minimize toxicity. CD3ε^−/−^ mice on the B10.A/SgSnAi background were from the NIAID Taconic Exchange. Prox1-CreER^T2^ and LtβR-floxed mice have been described previously [26], [27]. Ff/fl and fl/+ mice were treated with tamoxifen feed (Harlan) for 2 weeks. Animals were maintained in pathogen-free facilities.

### Isolation of Stromal Cells from LN and Peripheral Tissues

Brachial, axillary, inguinal, and mesenteric LN were harvested and pooled for all experiments involving LEC from adult mice, and for co-culture experiments involving postnatal day 7 mice. Only brachial LN were harvested on postnatal day 1 and postnatal day 7 for PD-L1 staining. Colon segments were excised and the lumen was washed with 1X PBS before mincing. Diaphragm was dissected, leaving out the central tendon. Minced tissue was digested in 0.42U/ml Liberase TM (Roche) and 40ug/ml DNAse (Sigma) for 40 minutes. Every 10 minutes, supernatant was pipetted into a collection tube and digestion media was replenished. LNSC were negatively selected with CD45 magnetic beads (Miltenyi) using the AutoMACS DepleteS program, and were >90% pure by flow cytometry.

### Flow Cytometry and Antibodies

Single cell suspensions of stromal cells were incubated with anti-CD16/CD31 blocking mAb (2.4G2, Bio Xcell) and then with antibodies against gp38 (BioLegend) and CD31 (eBioscience). Additional antibodies included those specific for CD45, PD-L1 (both from BioLegend), Lyve-1, ICAM-1, MAdCAM-1, and LtβR (all from eBioscience). LNSC were stained with Dapi to discriminate live and dead cells. For experiments involving the co-culture or transfer of FH T cells, Thy1.2 (eBioscience), CD45.1 (eBioscience), or Tyr_369_-tetramer were used as identifying markers. Flow cytometry was performed on a FACSCanto II (BD Biosciences) and data analyzed using FloJo software (Tree Star).

### Quantitative PCR

LNSC and LEC and BEC from diaphragm and colon were electronically sorted into RNA Protect Cell Reagent (Qiagen). mRNA was purified using the RNEasy Micro Kit (Qiagen). cDNA was synthesized using the iScript cDNA Synthesis Kit (Biorad). Amplification was performed using iQ SYBR Green Supermix (Biorad) and Ct values were detected using the iCycler iQ Real Time PCR Machine (Biorad).

### Microarray and Data Analysis

LEC and BEC from LN were purified as described above. mRNA was purified using the Arcturus PicoPure RNA Isolation Kit (Life Technologies) and quantified using the NanoDrop 2000 (Thermo Scientific). Purified mRNA underwent 2 round of linear amplification using the Arcturus RiboAmp HS Plus Kit (Life Technologies). Amplified RNA was biotin-labeled using the Arcturus Turbo Labeling Kit (Life Technologies) and hybridized to GeneChip Mouse Genome 430 2.0 Array chips (Affymetrix). 3 independent samples were analyzed. Pairwise reproducibility plots were performed for quality control. Those arrays that passed this quality control filter were normalized to arrive at relative log2 RNA abundance estimates using GCRMA within Bioconductor. Differentially expressed genes in LEC vs. BEC were determined at a 5% False Discovery Rate (FDR) using the *limma* package in Bioconductor. Microarray data has been deposited in the Gene Expression Omnibus (GEO) database with the accession number GSE53686.

### Immunofluorescence Microscopy

LN, diaphragm, and colon were placed in O.C.T. compound (Tissue Tek) and frozen on dry ice. Blocks were cut into 5 µm sections on Superfrost/Plus slides (Fisher). Tissue sections were fixed in ethanol and acetone at a 1∶1 ratio, blocked in 3% BSA-1X PBS containing 10% donkey serum and Fc block (2.4G2). Sections were stained with biotin-anti-PD-L1 (BioLegend), Alexa488- or eFluor660-anti-Lyve1, eFluor450-anti-B220, eFluor450-anti-CD31, FITC- or biotin-anti-MAdCAM-1 (all from eBioscience). Secondary reagent used was Streptavidin-Dylight594 (Jackson Immunoresearch). Images were taken using an Axio Imager 2 with Apotome (Carl Zeiss), and modified by adjusting brightness and contrast to the same levels (Adobe photoshop). Using ImageJ 1.46 software, we established a threshold signal to define Lyve-1^+^ pixels and a selection gate was created for each LN location. This gate was then transposed onto PD-L1 or MAdCAM-1 images of the same slide and the MFI for these two markers was calculated for the Lyve-1^+^ pixel distribution.

### Presentation of Tyr369 Epitope

Naïve FH T cells were positively selected with anti-CD8α magnetic beads (Miltenyi) using an AutoMACS (PosselS program) and labeled with Cell Trace Violet (CTV, Invitrogen) or CFSE. For in vivo experiments, 1 × 10^6^ Thy1.2^+^ or CD45.1^+^ FH and control Thy1.1^+^ or CD45.2^+^ CD8 T cells were adoptively transferred i.v. into WT or µMT^−/−^ AAD^+^ tyrosinase^+^ recipients. At 3 and 7 days post-transfer, peripheral LN were harvested, homogenized, and stained for CD8 and Thy1.2, CD45.1, or Tyr_369_-tetramer, and assessed for CTV dilution. For in vitro experiments, LNSC were liberated from LN, colon, and diaphragm from AAD^+^ tyrosinase^+^ mice as described above and LEC were electronically sorted into subpopulations (FACSVantage, Becton Dickinson or Reflection, iCyt). LEC were co-cultured with CFSE-labeled FH cells at a 1∶2 ratio in the presence of 10 U/ml IL-2 for 86 h and assessed for CFSE dilution. Peptide-pulsed LEC were prepared by incubation at 37°C for 1hr with 10 µg/ml Tyr_369_ peptide and washing twice.

### LtβR Blockade Experiments

C57BL/6 mice were treated with 100 µg LtβR-Ig [28] i.p. every 5 days for 1 or 4 weeks. Peripheral and mesenteric LN were pooled for LNSC enrichment for cell surface and tyrosinase gene expression analysis.

## Results

### LN-resident Lymphatic Endothelial Cells Exhibit Greater Tolerogenic Potential than Peripheral Tissue LEC

**Figure 1:**
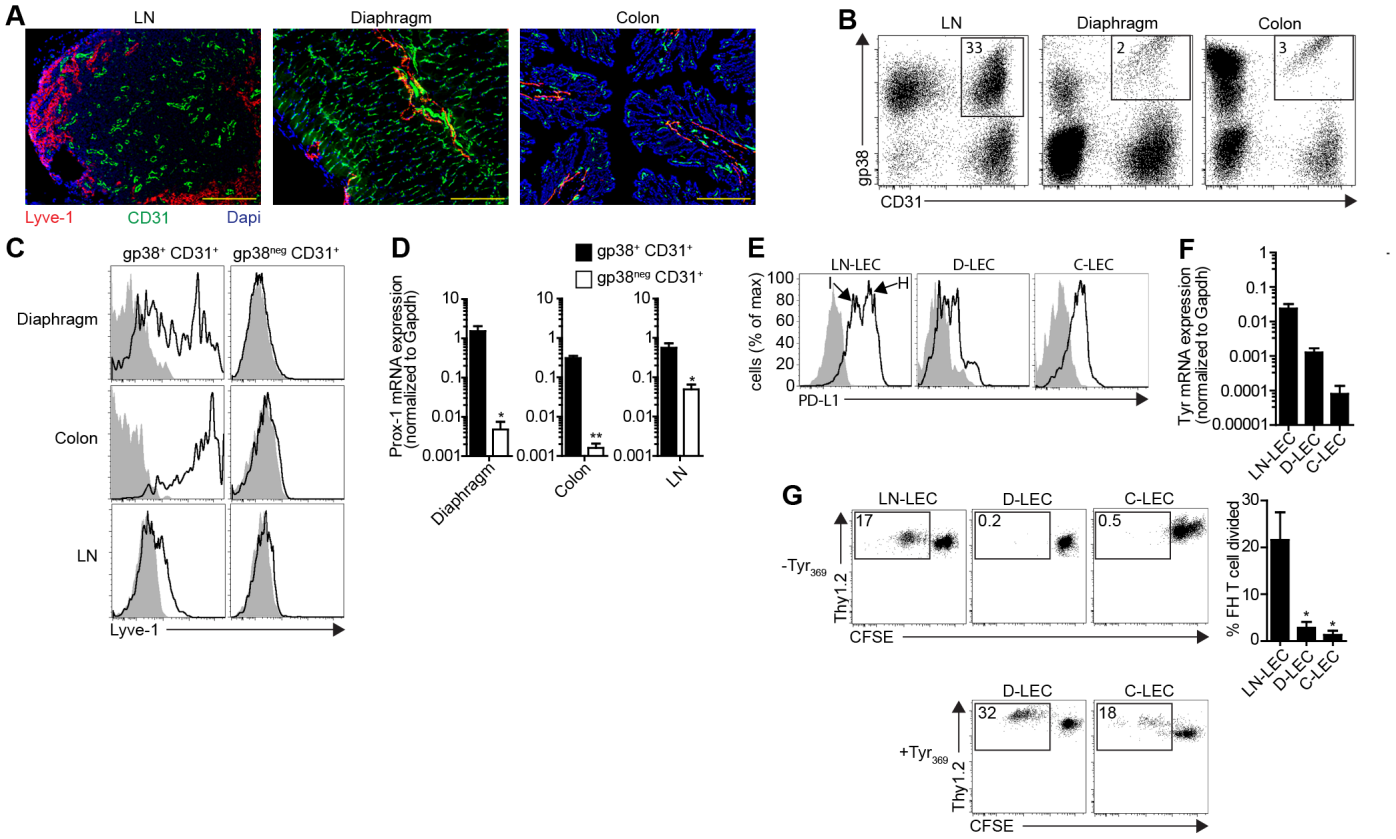
Immunological characteristics of LN-resident and peripheral tissue LEC. ***a.*** LN, diaphragm, and colon frozen sections were stained with antibodies specific for Lyve-1 and CD31. Dapi indicates nuclear staining. Scale bar = 200 µm. ***b.*** Single cell suspensions from the indicated tissues were stained with antibodies specific for CD45, gp38, and CD31, and analyzed by flow cytometry. Plots are gated on CD45^neg^ cells. Numbers represent percentage of total CD45^neg^ cells. ***c.*** Single cell suspensions were stained with antibodies specific for CD45, gp38, CD31, and Lyve-1. Plots are gated on CD45^neg^ cells and the indicated markers. ***d.*** CD45^neg^ gp38^+^ CD31^+^ and CD45^neg^ gp38^neg^ CD31^+^ cells from the indicated tissues were sorted electronically. mRNA was purified and qPCR was performed for Prox-1. Data are represented as mean +/− SEM. ***e.*** Single cells suspensions were stained with antibodies specific for CD45, gp38, CD31, and PD-L1. Plots are gated on of CD45^neg^ gp38^+^ CD31^+^ cells. MFI for LN-LEC^hi^ (H)  = 5434, LN-LEC^int^ (I)  = 1226, D-LEC  = 126, C-LEC  = 330; These MFI are net of background MFI, which is indicated by gray shading. ***f.*** CD45^neg^ gp38^+^ CD31^+^ LEC were purified from LN (LN-LEC), diaphragm (D-LEC), and colon (C-LEC) by electronic cell sorting. RNA was purified and qPCR was performed for tyrosinase. Data are represented as mean +/− SEM. ***g.*** LN-LEC, D-LEC, and C-LEC were purified by electronic cell sorting and either left un-pulsed (-Tyr_369_) or pulsed with Tyr_369_ peptide (+Tyr_369_). They were then co-cultured with Thy1.2^+^ CFSE-labeled, naïve FH T cells for 86hr and stained with antibodies specific for CD8 and Thy1.2. Representative plots are gated on CD8^+^ cells. Numbers represent percentage of divided FH T cells of total FH T cells. Summary of 3 independent experiments for the un-pulsed LEC co-culture group is shown. Data are represented as mean +/− SEM. *p<0.05.

Murine LEC in LN directly express *tyrosinase* mRNA, present the tyrosinase-derived epitope Tyr_369_, and induce deletion of tyrosinase-specific T cells via PD-L1 [10], [15]. To determine whether LEC that compose the lymphatic vasculature in non-lymphoid tissues had similar capabilities, LEC in the diaphragm and colon were identified by immunofluorescent staining for the LEC-specific marker, Lyve-1 [15], and BEC were identified by staining for CD31 (Fig. 1a). After collagenase digestion of these tissues to produce single cell suspensions [29], [30], gp38^+^ CD31^+^ and gp38^neg^ CD31^+^ subpopulations were identified among CD45^neg^ cells (Fig. 1b). The gp38^+^ CD31^+^ cells expressed Lyve-1 strongly, albeit heterogeneously, while the gp38^neg^ CD31^+^ cells, representing BEC, were uniformly negative (Fig. 1c). By quantitative RT-PCR (qPCR), the expression of *Prox-1*, the master transcriptional regulator of the LEC lineage, was substantially higher in purified gp38^+^ CD31^+^ cells than in gp38^neg^ CD31^+^ cells from both peripheral tissues (Fig. 1d), consistent with their identity as LEC.

To address the ability of peripheral tissue LEC to induce tolerance, we examined their expression of PD-L1 and tyrosinase in comparison to LEC isolated from major peripheral and mesenteric LN (LN-LEC). By flow cytometry two subpopulations of LN-LEC were distinguished that expressed intermediate and high levels of PD-L1 (PD-L1^int^, PD-L1^hi^, respectively). LEC from diaphragm (D-LEC) expressed ∼10 and 43-fold less, and LEC from colon (C-LEC) ∼4- and 16-fold less PD-L1, than the PD-L1^int^ and PD-L1^hi^ LN-LEC subpopulations, respectively (Fig. 1e). By qPCR, purified D-LEC and C-LEC expressed 17- and 250-fold lower levels of *tyrosinase* than LN-LEC, respectively (Fig. 1f). To assess whether this was sufficient for Tyr_369_ epitope presentation, FH T cells were co-cultured with purified LEC populations. Whereas LN-LEC induced strong proliferation, D-LEC and C-LEC did not (Fig. 1g). However, D-LEC and C-LEC that had been pulsed with Tyr_369_ peptide induced proliferation comparable to that induced by LN-LEC. Thus, peripheral tissue LEC express very low levels of PD-L1 that likely compromise their ability to induce T cell deletion, and do not express tyrosinase at levels sufficient to induce T cell proliferation.

**Figure 2:**
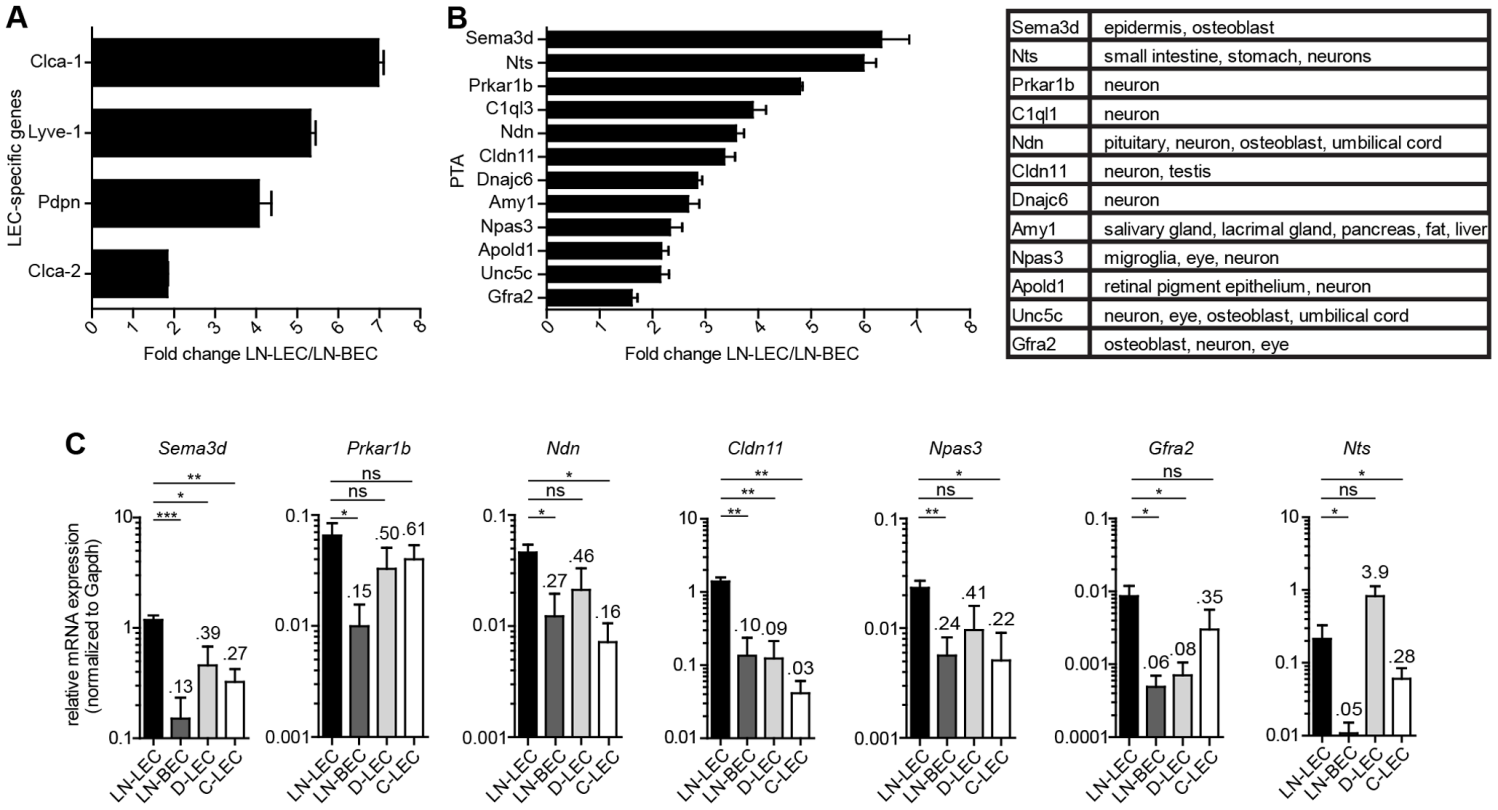
LN-LEC express PTA at higher levels than peripheral tissue LEC. ***a,b.*** LEC and BEC were purified from pooled pLN and mesLN by electronic cell sorting based on differential expression of gp38 and CD31. RNA was purified and amplified for microarray hybridization. Fold change represent the average of 3 independent experiments. PTA were identified as genes that were non-endothelial and non-hematopoietic, and expressed in five tissues or less as analyzed from the BioGPS (www.biogps.org). Data are represented as mean +/− SEM. All LEC-specific genes and PTA in LN-LEC samples had p<0.05 compared to LN-BEC samples. ***c.*** CD45^neg^ gp38^+^ CD31^+^ LEC from LN (LN-LEC), diaphragm (D-LEC), and colon (C-LEC), as well as CD45^neg^ gp38^neg^ CD31^+^ BEC from LN (LN-BEC) were electronically sorted, mRNA purified, and qPCR performed for the indicated genes. Numbers indicate average fold change compared to LN-LEC. Data are represented as mean +/− SEM. *p<0.05, **p<0.01, ***p<0.001, ns = not significant.

We next addressed whether differential expression of tyrosinase by LN-LEC and peripheral tissue LEC was generalizable to additional PTA. PTA have been identified in thymic epithelial cells and LN eTACs based on microarray comparisons of cells from Aire positive and negative mice [31]. However, LEC and FRC in LN are Aire negative [10] and the transcription factors controlling PTA expression in these cells have not been identified. Because the number of PTA known to be expressed by LEC is very small [10], [11], we performed a microarray analysis to compare the transcriptional profiles of purified LN-LEC and LN-BEC. We reasoned that because these two endothelial cell lineages are developmentally related [32], genes shared between them are likely to be important to endothelial cell function, and less likely to be PTA, and that genes differentially expressed by LN-LEC include PTA. We identified 221 genes that were more highly expressed by LN-LEC than LN-BEC, some of which were specific to the LEC lineage (Fig. 2a). In accordance with criteria used in thymus [33], we defined PTA as genes expressed in 5 or less tissues using a reference database [34]. To avoid possible cross-contamination, we also excluded genes expressed by hematopoietic cells. Out of the 221 genes overexpressed by LN-LEC, 12 fit these criteria (Fig. 2b). Further analysis of 7 of these by qPCR confirmed that all were more highly expressed by LN-LEC than LN-BEC (Fig. 2c). Six of 7 were also expressed at considerably lower levels in either D-LEC or C-LEC or both (Fig. 2c). Collectively, these results demonstrate that the LN microenvironment controls high PD-L1 and tyrosinase expression by LEC and likely controls global expression of PTA.

### Definition of Distinct LN-LEC Subpopulations that Vary in their Location and Expression of Tyrosinase

**Figure 3:**
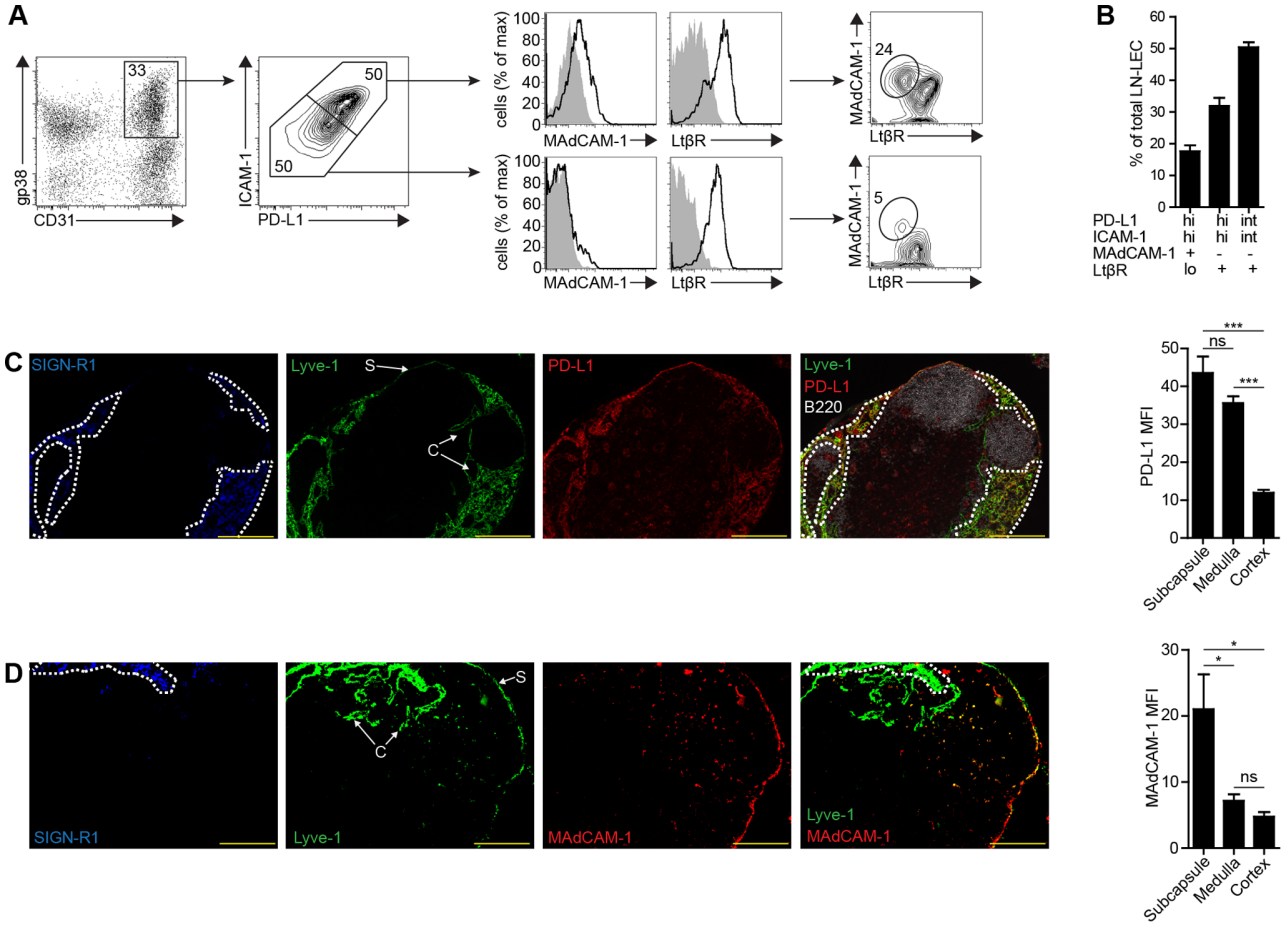
PD-L1 in combination with ICAM-1, MAdCAM-1, and LtβR define distinct LEC subsets that vary in location in the LN. ***a.*** Single cell suspensions of CD45^neg^ LN cells were stained with antibodies against PD-L1, ICAM-1, MAdCAM-1, and LtβR. Number in the gp38 vs. CD31 2-D plot indicates percentage of total CD45^neg^ cells. Number in the 2-D PD-L1 vs. ICAM-1 plot indicates percentage of total LEC. Number in the 2-D MAdCAM-1 vs. LtβR plots indicates percentage of total PD-L1^hi^ ICAM-1^hi^ LEC (top) or PD-L1^int^ ICAM-1^int^ LEC (bottom). ***b.*** Summary graph of 7 independent experiments of the indicated LEC subpopulation percentage of total LEC. Data are represented as mean +/− SEM. ***c, d.*** Frozen axillary LN sections were stained with antibodies specific for SIGN-R1, PD-L1, Lyve-1, CD31, and B220 (c) or SIGN-R1, MAdCAM-1, and Lyve-1 (d). Dashed line represents regions of SIGN-R1 staining. S = subcapsule, C = cortex. Scale bar = 200 µm. Staining is representative of multiple fields from 3 experiments consisting of 2 separate LN from 3 mice. Graphs represent PD-L1 (c) or MAdCAM-1 (d) MFI gated on Lyve-1^+^ pixels in the indicated LN compartments. Data are represented as mean +/− SEM. *p<0.05, ***p<0.001, ns = not significant.

The existence of LN-LEC expressing either intermediate or high levels of PD-L1 (Fig. 1c) led us to question whether they might represent subpopulations that also varied in their differentiation status and/or location. LN-LEC also expressed intermediate or high levels of ICAM-1, and the levels of PD-L1 and ICAM-1 were correlated (Fig. 3a). While most LN-LEC expressed lymphotoxin β receptor (LtβR), a small fraction of PD-L1^hi^ ICAM-1^hi^ LN-LEC did not (Fig. 3a). However, this subset was the only LN-LEC population that expressed MAdCAM-1. Thus, three distinct LN-LEC subpopulations can be discriminated based on differential expression of these markers: PD-L1^hi^ ICAM-1^hi^ MAdCAM-1^+^ LtβR^lo^, PD-L1^hi^ ICAM-1^hi^ MAdCAM-1^neg^ LtβR^+^, and PD-L1^int^ ICAM-1^int^ MAdCAM-1^neg^ LtβR^+^. These represent roughly 20%, 30%, and 50% of LN-LEC, respectively (Fig. 3b).

To determine whether these LEC subpopulations were localized to distinct anatomical locations in the LN, we performed immunofluorescence microscopy on LN sections. SIGN-R1, a marker of medullary macrophages [35], was used to distinguish this area from the subcapsule and cortex. We considered thin layers of Lyve-1^+^ cells along SIGN-R1^neg^ outer edges of the LN to be subcapsular LEC, and Lyve-1^+^ cells that formed SIGN-R1^neg^ internal structures to be cortical LEC. We set a threshold value to define Lyve-1^+^ pixels and created a selection gate for each LN location. This gate was then transposed onto PD-L1 or MAdCAM-1 images of the same slide and the MFI of these markers was calculated. Strong PD-L1 staining was apparent on Lyve-1^+^ cells in the medulla and subcapsule, but was much weaker on those in the cortex (Fig. 3c). MAdCAM-1 staining co-localized exclusively with Lyve-1^+^ cells in the subcapsule (Fig. 3d). These data indicate that the PD-L1^hi^ ICAM-1^hi^ MAdCAM-1^+^ LtβR^lo^ subpopulation represents subcapsular LEC, the PD-L1^hi^ ICAM-1^hi^ MAdCAM-1^neg^ LtβR^+^ subpopulation represents medullary LEC, and the PD-L1^int^ ICAM-1^int^ MAdCAM-1^neg^ LtβR^+^ subpopulation represents cortical LEC.

**Figure 4:**
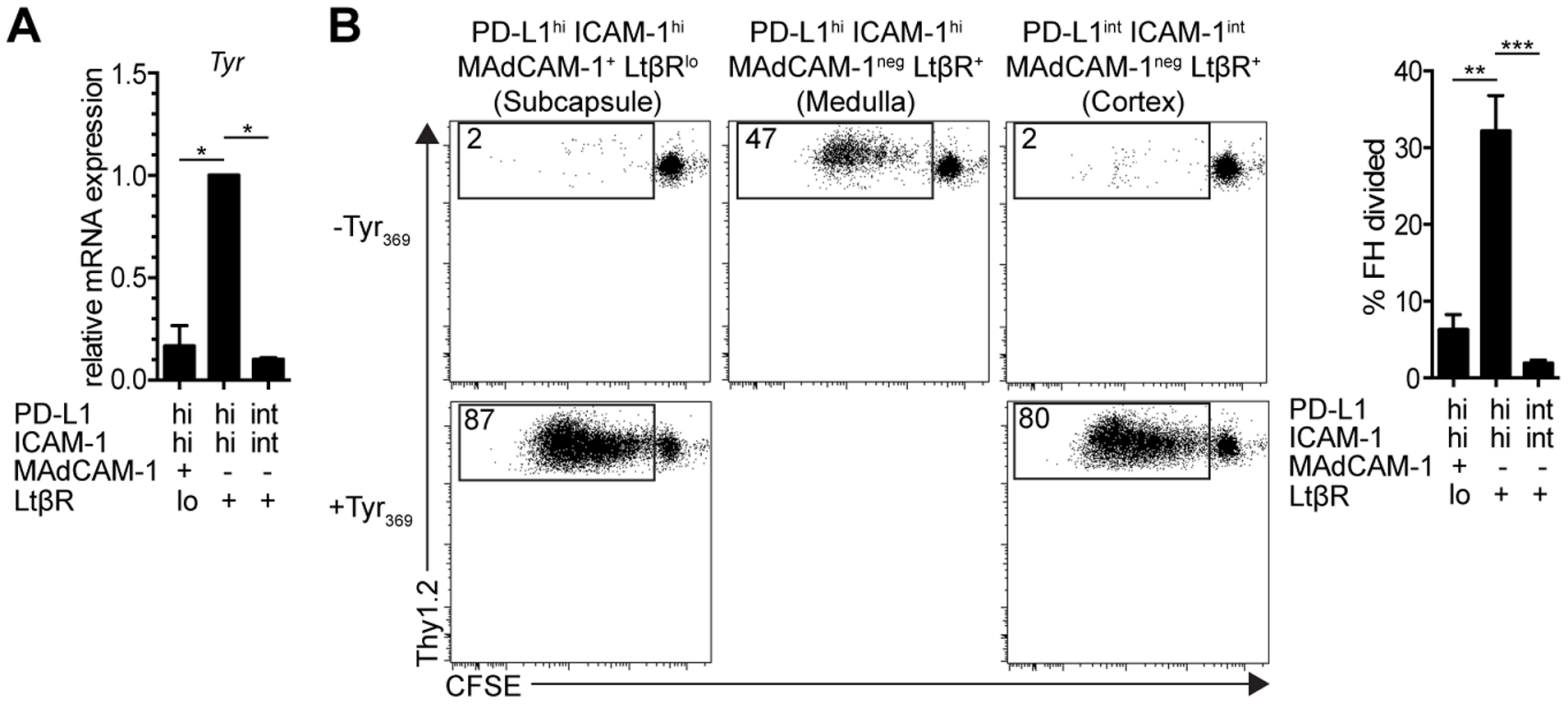
Tyr_369_ is presented exclusively by LEC that occupy the LN medulla. ***a.*** LNSC, obtained by enzymatic digestion and CD45 magnetic bead separation, were stained with antibodies against gp38 and CD31, as well as, PD-L1, ICAM-1, MAdCAM-1, and LtβR. LEC subpopulations were identified based on differential expression of these markers and were sorted electronically. mRNA was purified and qPCR for tyrosinase was performed. Ct values were first normalized to Gapdh (2^ΔCt^), and then normalized to the 2^ΔCt^ value of medullary sinus LEC. Data are representative of 3 independent experiments and are represented as mean +/− SEM. ***b.*** *Left panels*, representative FACS plots of CFSE-labeled naïve Thy1.2^+^ FH T cells co-cultured with cells of the indicated un-pulsed or Tyr_369_-pulsed purified LEC subpopulations for 86hr in the presence of IL-2. FH T cells were stained with antibodies against CD8 and Thy1.2; *right panel*, summary graph of 3 independent experiments of FH T cells co-cultured with un-pulsed LEC subpopulations. Data are represented as mean +/− SEM. *p<0.05, **p<0.01, ***p<0.001.

To determine whether these LN-LEC subpopulations also varied in tyrosinase expression, we electronically sorted them based on differential expression of PD-L1, ICAM-1, MAdCAM-1, and LtβR, and performed qPCR. PD-L1^hi^ ICAM-1^hi^ MAdCAM-1^neg^ LtβR^+^ cells representing medullary LEC expressed *tyrosinase* at a ∼10-fold higher level compared to the other two subpopulations (Fig. 4a). Consistent with this, PD-L1^hi^ ICAM-1^hi^ MAdCAM-1^neg^ LtβR^+^ LN-LEC induced robust proliferation of FH T cells when co-cultured in vitro (Fig. 4b), demonstrating presentation of the Tyr_369_ epitope. The other two subpopulations induced only a weak proliferative response, which was substantially increased when they were pulsed with exogenous Tyr_369_ peptide (Fig. 4b). These results strongly suggest that tolerance to tyrosinase is primarily induced by PD-L1^hi^ ICAM-1^hi^ MAdCAM-1^neg^ LtβR^+^ LEC that occupy the LN medulla.

### Lymphotoxin β Receptor Signaling Controls Expression of PD-L1 and MAdCAM-1 on LN-LEC, but Not Anatomical Distribution of LN-LEC Subpopulations

**Figure 5:**
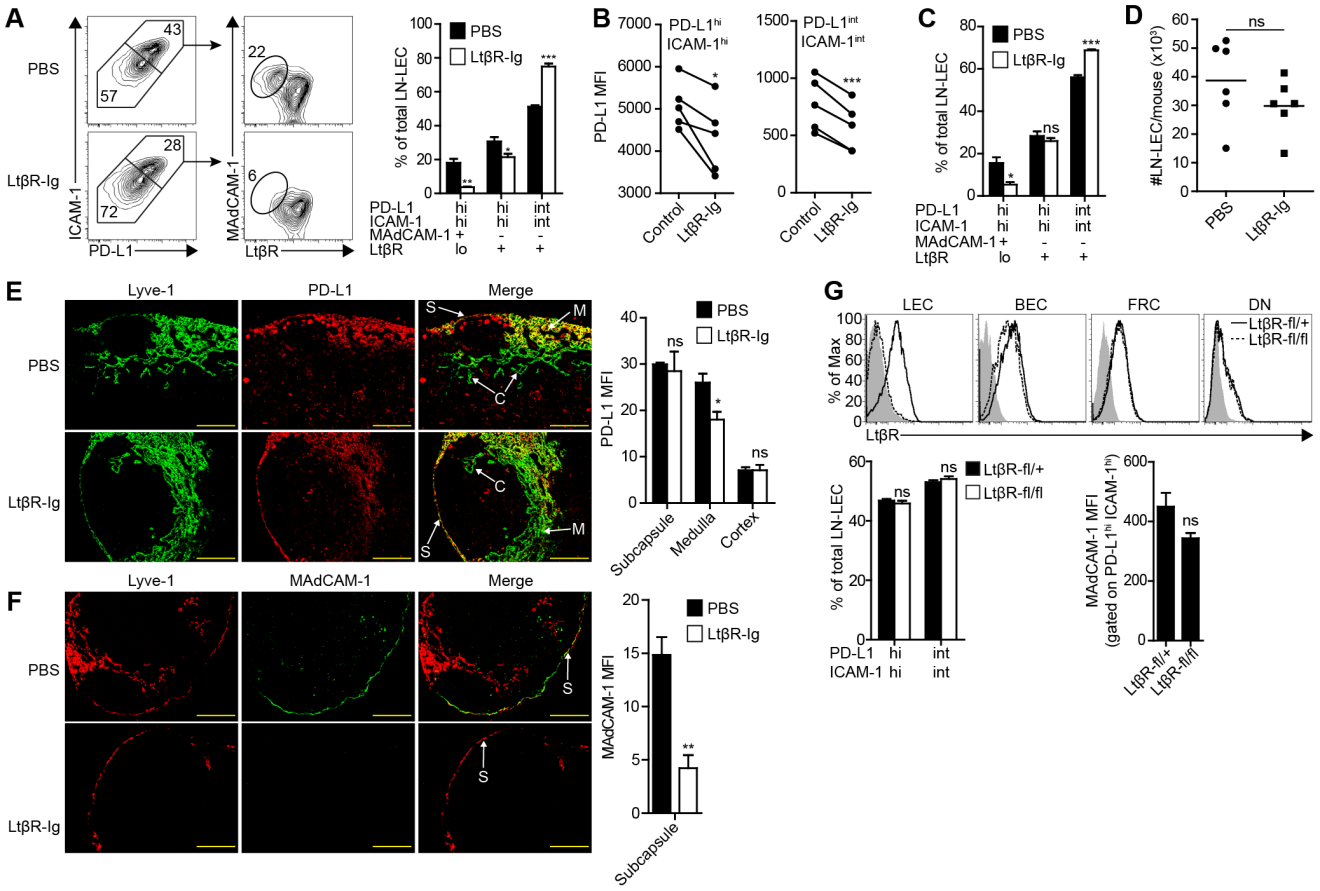
LtβR signaling is required for high level PD-L1 expression by medullary LEC, and MAdCAM-1 expression on subcapsular LEC. ***a.*** LNSC were purified by enzymatic digestion of pooled LN and CD45 magnetic bead separation from mice treated for 4 weeks with LtβR-Ig or PBS, and stained with antibodies specific for gp38, CD31, PD-L1, ICAM-1, MAdCAM-1, and LtβR, and analyzed by flow cytometry. *Left panel*, plots display data gated on CD45^neg^ gp38^+^ CD31^+^ LEC and subsequently gated on PD-L1 and ICAM-1 co-expressing subpopulations. Numbers indicate percentage of gated population out of total LEC (PD-L1 vs. ICAM-1 2-D plots), or out of the total PD-L1^hi^ ICAM-1^hi^ subpopulation (MAdCAM-1 vs. LtβR 2-D plots). *Right panel*, summary of 3 independent experiments. Data are represented as mean +/− SEM. ***b.*** PD-L1 MFI of PD-L1^hi^ ICAM-1^hi^ and PD-L1^int^ ICAM-1^int^ subpopulations gated on gp38^+^ CD31^+^ LN cells of the indicated mice. Each set of paired samples represents an independent experiment. ***c.*** LEC absolute number was calculated from cells that were gated as Dapi^neg^, singlets, CD45^neg^, gp38^+^, CD31^+^ cells by flow cytometry. Data are represented as mean +/− SEM. *d.* Analysis of gp38^+^ CD31^+^ LN cells as in (a) in mice treated for 1 week with LtβR-Ig or PBS. Data from 3 independent experiments. ***e.*** *Left panel*, frozen axillary LN sections of indicated mice were stained with antibodies specific for Lyve-1, and PD-L1. *Right panel*, summary plot of PD-L1 MFI gated on Lyve-1^+^ pixels in the indicated LN location. Data are represented as mean +/− SEM. Significance values represent a comparison of the MFI of PD-L1 staining of Lyve-1^+^ vessels between LtβR-Ig and PBS treated mice of the indicated LN compartment. S = subcapsule C = cortex, M = medulla. Scale bar = 200 µm. *f. Left panel*, frozen axillary LN sections from indicated mice were stained with antibodies specific for Lyve-1 and MAdCAM-1. Scale bar = 200 µm. *Right panel*, summary plot of MAdCAM-1 MFI gated on Lyve-1^+^ pixels in the LN subcapsule. MFI calculated as in (c). Staining is representative of multiple fields from 3 independent experiments consisting of 2 separate LN from 3 mice. Data are represented as mean +/− SEM. *p<0.05, **p<0.01, ***p<0.001, ns = not significant. *g.* Prox1-CreERT2 x LtβR^fl/+^ (LtβR-fl/+) and Prox1-CreERT2 x LtβR^fl/fl^ (LtβR-fl/fl) mice were treated for 2 weeks with a Tamoxifen diet. *Upper panel,* LNSC were purified as above, stained with antibodies specific for CD45, gp38, CD31, and LtβR and assessed by flow cytometry. *Lower panels*, summary plots of the representation of PD-L1 and ICAM-1 LN-LEC subpopulations and MAdCAM-1 MFI. Data are represented as mean +/− SEM. ns = not significant.

The observation that LN-LEC express PD-L1 and tyrosinase more highly than peripheral tissue LEC suggested that factors in the LN microenvironment control these tolerogenic properties. Tumor necrosis factor receptor (TNFR) family members control multiple aspects of LN development and maintenance of the phenotype of stromal cells in the adult LN [36]. No change in the representation of PD-L1-expressing LN-LEC subpopulations was observed in mice lacking TNFα (Fig. S1). However, in mice treated for 4 weeks with LtβR-Ig, which acts as a decoy receptor for LtβR ligands, there was a 2-fold decrease in the percentage of PD-L1^hi^ ICAM-1^hi^ LN-LEC, and a reciprocal increase in the PD-L1^int^ ICAM-1^int^ subpopulation (Fig. 5a). There was also a decrease in the PD-L1 MFI of the PD-L1^hi^ and PD-L1^int^ subpopulations (Fig. 5b). In addition, the percentage of PD-L1^hi^ ICAM-1^hi^ LEC that were MAdCAM-1^+^ LtβR^lo^ was substantially diminished (Fig 5a). A similar trend was also observed in mice that were treated with LtβR-Ig for 1 week (Fig. 5c). Despite these alterations, there was no change in the absolute number of LN-LEC (Fig. 5d), and their distribution in the subcapsular, cortical and medullary areas of LN from LtβR-Ig treated mice was normal (Fig. 5e,f). Thus, LN-LEC continued to occupy the subcapsule despite the lack of a MAdCAM-1^+^ LtβR^lo^ subpopulation (Fig. 5f). A quantitative analysis of PD-L1 staining intensity of Lyve-1^+^ pixels showed that average expression in the LN subcapsule and cortex was comparable in control and LtβR-Ig-treated mice (Fig. 5e). In contrast, average PD-L1 expression on Lyve-1^+^ cells in the medulla of LtβR-Ig-treated mice was significantly diminished, which is consistent with the decrease in PD-L1 MFI on PD-L1^hi^ ICAM-1^hi^ LEC (Fig. 5b). Interestingly, inducible deletion of LtβR specifically in LEC of adult mice did not alter PD-L1 or MAdCAM-1 expression (Fig. 5g). Thus, LtβR signaling acts via a secondary mechanism to control MAdCAM-1 expression on LEC in the subcapsule, full representation of PD-L1^hi^ ICAM-1^hi^ cells, and full expression of PD-L1 on LEC that occupy the LN medulla.

### B and T Cells Control Expression of PD-L1 and MAdCAM-1 on LN-LEC

DC and lymphocytes produce Ltα and Ltβ in secondary lymphoid organs under non-inflammatory conditions [37], [38], and Ltα_1_β_2_ derived from DC maintains PNAd expression on HEV [39]. To determine whether DC are the source of Ltβ that modulates PD-L1 expression by LN-LEC, we evaluated Batf3^−/−^ mice, which lack the CD8α^+^ and CD103^+^ DC subsets, and CD11c-DTR mice, in which all conventional DC subsets are ablated by diphtheria toxin (DT) administration. After 1 week of DT treatment, the representation and absolute numbers of CD11c^+^ DC in peripheral LN were significantly diminished (Fig. S2a). However, no changes in PD-L1 expression by LEC were observed in either these or Batf3^−/−^ mice (Fig. S2b,c).

**Figure 6:**
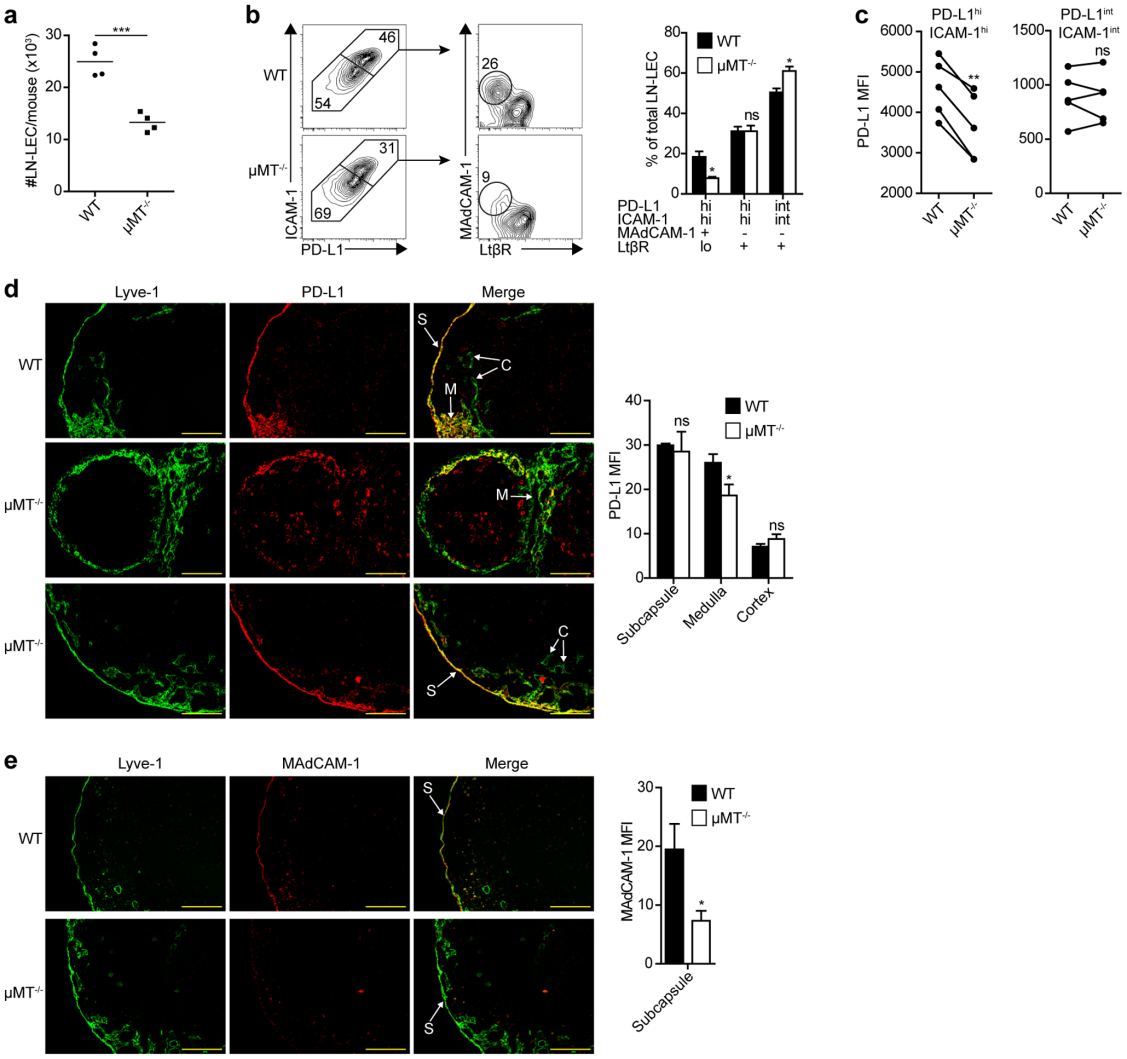
B cells control the representation and PD-L1 expression level of medullary LEC, and MAdCAM-1 expression on subcapsular LEC. ***a.*** LNSC were purified by enzymatic digestion of pooled LN and CD45 magnetic bead separation from µMT^−/−^ or WT mice. LEC absolute number was calculated from cells that were gated as Dapi^neg^, singlets, CD45^neg^, gp38^+^, CD31^+^ cells by flow cytometry. *b.* LNSC were stained with antibodies specific for gp38, CD31, PD-L1, ICAM-1, MAdCAM-1, and LtβR, and analyzed by flow cytometry. *Left panel*, plots display data gated on CD45^neg^ gp38^+^ CD31^+^ LEC and subsequently gated on PD-L1 and ICAM-1 co-expressing subpopulations. Numbers indicate percentage of gated population out of total LEC (PD-L1 vs. ICAM-1 2-D plots), or out of total PD-L1^hi^ ICAM-1^hi^ or PD-L1^int^ ICAM-1^int^ subpopulations (MAdCAM-1 vs. LtβR 2-D plots). *Right panel*, summary of 3 independent experiments. Data are represented as mean +/− SEM. *c.* PD-L1 MFI of PD-L1^hi^ ICAM-1^hi^ and PD-L1^int^ ICAM-1^int^ subpopulations gated on CD45^neg^ gp38^+^ CD31^+^ LN cells of the indicated mice. Each set of paired samples represents an independent experiment. *d. Left panel*, frozen axillary LN sections of indicated mice were stained with antibodies specific for Lyve-1, and PD-L1. *Right panel*, summary plot of PD-L1 MFI of Lyve-1^+^ pixels in the indicated LN location. Data are represented as mean +/− SEM. Significance values represent a comparison of the MFI of PD-L1 staining of Lyve-1^+^ vessels between LtβR-Ig and PBS treated mice of the indicated LN compartment. S = subcapsule C = cortex, M = medulla. Scale bar = 200 µm. *e. Left panel*, frozen axillary LN sections from indicated mice were stained with antibodies specific for Lyve-1 and MAdCAM-1. Scale bar = 200 µm. *Right panel*, summary plot of MAdCAM-1 MFI of Lyve-1^+^ pixels in the LN subcapsule. Data are represented as mean +/− SEM. MFI calculated as in (c). Staining is representative of multiple fields from 3 independent experiments consisting of 2 separate LN from 3 mice. *p<0.05, **p<0.01, ***p<0.001, ns = not significant.

To test the hypothesis that lymphocytes control the phenotypes of LN-LEC we evaluated µMT^−/−^, CD3ε^−/−^ and Rag1^−/−^ mice. In all of these mice, the absolute number of LN-LEC was significantly less than in wild type mice (Fig. 6a, S3a, data not shown), consistent with an overall decrease in LN size. Similar to what was observed in LtβR-Ig treated mice, the representation of PD-L1^hi^ ICAM-1^hi^ MAdCAM-1^+^ LtβR^lo^ LEC was considerably diminished in both µMT^−/−^ and Rag1^−/−^ mice, while the representation of PD-L1^int^ ICAM-1^int^ MAdCAM-1^neg^ LtβR^+^ LEC was increased (Fig. 6b, data not shown). Although there was no change in the representation of PD-L1^hi^ ICAM-1^hi^ MAdCAM-1^neg^ LN-LEC, the MFI of PD-L1 expression was significantly lower (Fig 6c). Interestingly, these trends in the representation of LEC subpopulations were reversed in CD3ε^−/−^ mice, while the PD-L1^hi^ ICAM-1^hi^ and PD-L1^int^ ICAM-1^int^ LEC subpopulations trended toward an increase in PD-L1 MFI (Fig. S3b,c). By immunofluorescence microscopy, PD-L1 expression on Lyve-1^+^ cells in the LN subcapsule and cortex of µMT^−/−^ mice was comparable to that in wild type mice, while those in the medulla expressed significantly less (Fig. 6d). In keeping with the flow cytometry analysis, Lyve-1^+^ cells in the LN cortex of CD3ε^−/−^ mice expressed significantly more PD-L1 on average (Fig. S3d). Lyve-1^+^ cells in the LN subcapsule of µMT^−/−^ mice expressed significantly less MAdCAM-1 on average (Fig. 6e), similar to what was observed in LtβR-Ig treated mice. However, MAdCAM-1 staining in CD3ε^−/−^ mice extended to Lyve-1^+^ cells in the medulla, as well as BEC (Fig. S3e). These results demonstrate that B cells are required for MAdCAM-1 expression on LEC in the subcapsule, and for full expression of PD-L1 on PD-L1^hi^ ICAM-1^hi^ cells that occupy the medulla. Their effects are largely the same as those mediated by indirect LTβR signaling. Conversely, T cells directly or indirectly suppress PD-L1 and MAdCAM-1 expression on LEC subpopulations by an unknown mechanism.

### Representation of Medullary Sinus LEC that Express High Level PD-L1 Alters the Deletion Kinetics of FH T Cells

**Figure 7:**
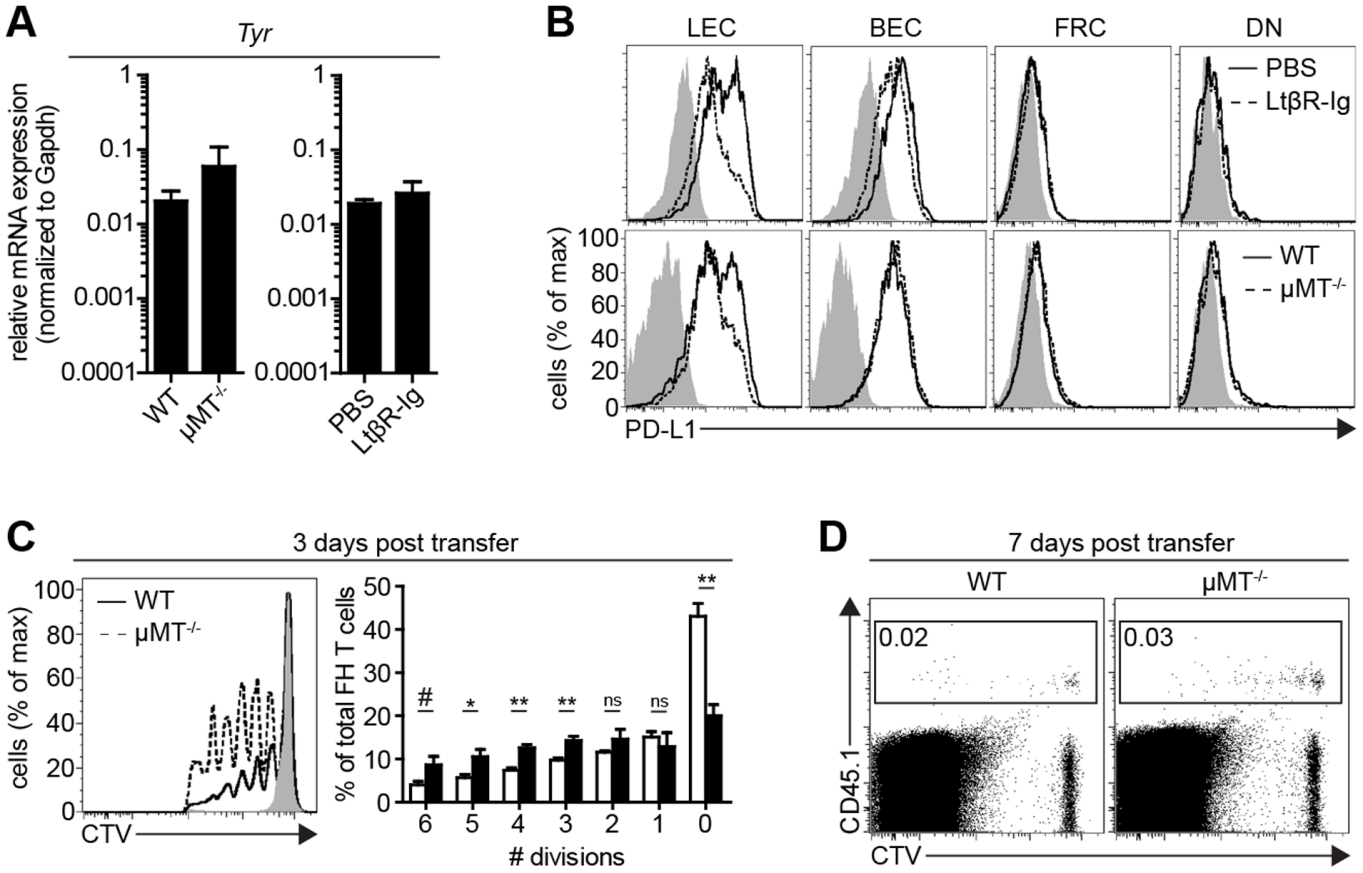
LtβR-Ig signaling and B cells do not control tyrosinase expression but influence the kinetics of FH T cell deletion. ***a.*** LEC were purified from the LN of mice treated with PBS or LtβR-Ig for 4 weeks, or from WT (C57BL/6) or µMT^−/−^ mice. RNA was purified and 40-cycle qPCR was performed for tyrosinase. Graphs represent results from 2 independent experiments. Data are represented as mean +/− SEM. ***b.*** LNSC were purified by enzymatic digestion of pooled LN from the indicated mice, obtained via magnetic bead separation, and were analyzed by flow cytometry. Plots are gated on CD45^neg^ gp38^+^ CD31^+^ cells. ***c.*** 1 × 10^6^ CD8-enriched FH T cells were labeled with cell trace violet (CTV)-labeled and adoptively transferred into WT, µMT^−/−^, or Tyr^neg^ (albino) recipients. At day 3 post-transfer, pooled peripheral LN were harvested and stained for CD8 and Tyr_369_-tetramer, and assessed for CTV dilution. *Left panel*, representative histogram plot gated on CD8^+^ Tet^+^ cells. *Right panel,* summary graph of percent FH T cells divided per division representative of 4 independent experiments. Data are represented as mean +/− SEM. *p<0.05, **p<0.01, ^#^p = 0.06 ns = not significant. ***d.*** Experiment performed as in (c), except FH T cells were identified using CD45.1 and LN were harvested at 7 days post transfer.

We next determined whether LtβR signaling and B cells control tyrosinase expression. By qPCR, the level of tyrosinase expression did not substantially differ among LEC purified from LN of LtβR-Ig treated, µMT^−/−^, or control mice (Fig. 7a). Since LN-LEC are the only LNSC subset that shows diminished PD-L1 expression in LtβR-Ig treated or µMT^−/−^ mice (Fig. 7b), we assessed whether this altered the development of FH T cell peripheral tolerance. FH T cell transfer experiments were performed in µMT^−/−^ mice, since LtβR-Ig treatment reduces PNAd^+^ HEV [40] and could prevent transferred cells from accessing the LN. At 3 days post-transfer, there was a higher representation of FH T cells in later cell divisions in the LN of µMT^−/−^ mice compared to wild type mice (Fig. 7c). However, by day 7, FH T cells in both types of mice were almost entirely eliminated (Fig. 7d). Thus, a diminished representation of medullary sinus LEC that express high level PD-L1 resulted in a delay in deletional tolerance.

### LN-LEC do not Mediate Tolerance to Tyrosinase in the Neonatal Period

**Figure 8:**
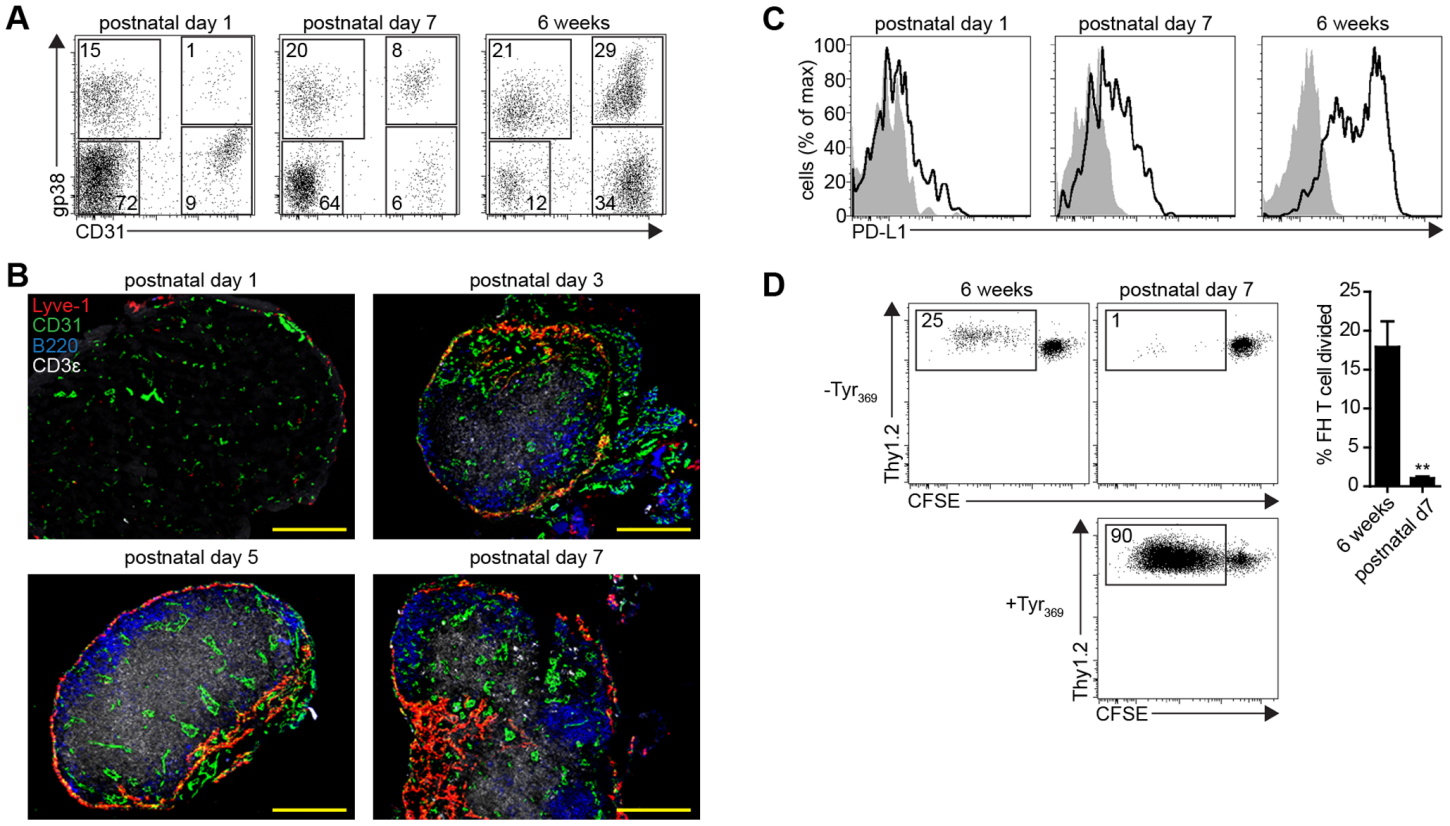
LN-LEC do not mediate tolerance to tyrosinase in the neonatal period. ***a.*** Brachial LN were purified from mice at the indicated age and enzymatically digested. For postnatal day 7 and 6 week old mice, LNSC were purified by CD45 magnetic bead separation. Single cell suspensions were stained with antibodies specific for CD45, gp38 and CD31. Numbers in quadrants to the right of plots represent the percentage out of CD45^neg^ cells. ***b.*** Frozen brachial LN sections from mice of the indicated age were stained with antibodies specific for Lyve-1, CD31, B220, and CD3ε. Scale bar = 200 µm. ***c.*** LNSC from the brachial LN of the indicated mice were stained with antibodies specific for CD45, gp38, CD31, PD-L1. Plots are gated on CD45^neg^ gp38^+^ CD31^+^ cells. ***d.*** Proliferation of CFSE-labeled naïve Thy1.2^+^ FH T cells co-cultured with the indicated Tyr_369_ pulsed, or un-pulsed LN-LEC population after 86hr. Numbers indicate the percentage of divided FH T cells of total FH T cells. *Left panels*, representative experiment; *right panel*, summary data for 2 independent experiments. **p<0.01.

The lymphatic vasculature differentiates from BEC precursors in the anterior cardinal vein at embryonic day 9.5 [17], and the LN anlage is formed when clusters of mesenchymal cells protrude into some of these primitive lymphatic vessels [41]. Thus, peripheral tissue LEC and LN-LEC stem from a common precursor LEC pool. Since these LEC populations differ significantly in expression of PD-L1 and tyrosinase, we wanted to determine the time frame in which the tolerogenic phenotype of LN-LEC developed. Neonatal LN were enzymatically digested and CD45^neg^ cells were enriched and stained for gp38 and CD31 to identify LEC and other LNSC populations. LEC represent ∼1% of LNSC on postnatal day 1 and ∼8% on postnatal day 7, considerably less than the ∼30% typical of 6 week old and older mice (Fig. 8a). Immunofluorescence imaging also established that LEC are restricted to the LN subcapsule on postnatal days 1, 3, and 5, but form a presumptive medullary region by postnatal day 7 (Fig. 8b). Only a small fraction of LN-LEC express PD-L1 above background levels at postnatal day 1. By postnatal day 7, some LN-LEC express PD-L1 at a level similar to that of PD-L1^int^ LN-LEC in 6 week old mice (Fig. 8c). Interestingly, B and T cell LN infiltration was evident at postnatal day 3. However, FH T cells did not proliferate when co-cultured with LN-LEC from postnatal day 7 mice (Fig. 8d). These results demonstrate that the ability of LN-LEC to induce tolerance to tyrosinase is developmentally regulated during the neonatal period, based on expression of both tyrosinase and PD-L1.

## Discussion

In this study, we investigated the spatial characteristics of LEC-mediated peripheral tolerance and the mechanisms by which the LN microenvironment endows LEC with the ability to induce CD8 deletional tolerance to tyrosinase. We demonstrated that LEC in peripheral tissues express considerably less PD-L1 and PTA than LEC in LN. Importantly, the level of tyrosinase expressed by LEC in peripheral tissues is insufficient to induce proliferation of FH T cells, suggesting that cells and factors in the LN microenvironment control the tolerance-inducing properties of LEC. Within the LN, we identified subsets of LEC with different anatomical locations, and found that high-level expression of tyrosinase and Tyr_369_ presentation is restricted to LEC that occupy the medulla. Furthermore, while LtβR signaling and B cells did not control tyrosinase expression, they did control expression of MAdCAM-1 on LEC in the subcapsule, the representation of LEC that expressed high level PD-L1 in the medulla, and the maximum level of PD-L1 expression on those cells, which influences the deletional kinetics of FH T cells. Finally, we demonstrated that the development of characteristics that enable LEC to induce tolerance to tyrosinase occurs after the neonatal period.

Thus far, LEC, FRC, and eTAC have been shown to express PTA and mediate peripheral tolerance in secondary lymphoid organs [9]–[11]. However, although eTAC do not reside in peripheral tissues, the direct tolerogenic potential of gp38^+^ CD31^neg^ cells or LEC in peripheral tissues had not been previously investigated. Our results suggest that tolerance induction is not a major function of LEC in peripheral tissues. We found that PD-L1 expression on LEC from the diaphragm is only slightly above background, and on only a small fraction of cells. LEC from colon express somewhat higher levels, but still well below the level on PD-L1^int^ LN-LEC. The level of PD-L1 expressed by D-LEC is comparable, while that of C-LEC is higher than that expressed by FRC. Almost all PTA identified by microarray analysis are expressed at lower levels by either D-LEC and C-LEC than by LN-LEC, and neither D-LEC nor C-LEC express sufficient tyrosinase to activate FH T cells. The relationship between mRNA expression and epitope display is influenced by protein translation, folding, and antigen processing, and epitopes from some PTA may be expressed by peripheral tissue LEC at levels that could engage self-reactive T cells. Based on the low level of PD-L1 by these LEC, it is unlikely this would result in direct deletion. However, as these activated self-reactive T cells travel to the LN, they would have the opportunity to interact with LN-LEC that express higher levels of both Ag and PD-L1.

It has long been appreciated that LEC form lymphatic sinuses in 3 distinct locations in LN. Here, we demonstrate that LEC subpopulations in these locations can be distinguished based on differences in PD-L1, ICAM-1, MAdCAM-1, and LtβR expression. This provides a means to isolate these cells for further analyses, which has been recognized as a limitation in earlier work [42]. We used this classification to evaluate the factors controlling the development of these subpopulations and their ability to induce immunological tolerance. We previously demonstrated that LN-LEC present Tyr_369_ to FH T cells [10] and that FH T cell deletion depends on engagement of PD-L1 on a radioresistant LNSC [15]. Here we showed that Tyr_369_ presentation is confined to LEC that occupy the medulla, and these cells express a higher level of PD-L1 than those in the cortex. Also, FH T cells delete with delayed kinetics in µMT^−/−^ mice, in which the representation of medullary LEC that express a high level of PD-L1 is reduced, as is the level of PD-L1 expression on those cells. This is consistent with a model in which naïve T cells engage Ag as they attempt to leave the LN. While FH T cells undergo 2–3 divisions before up-regulating PD-1 [15], it is well established that Ag engagement suppresses expression of the sphingosine phosphate receptor S1P1, which normally mediates egress into efferent lymphatics [24]. Thus, Ag-activated FH T cells might remain in the LN and engage PD-L1 on the same Ag-expressing medullary LEC. However, it is also possible that FH T cells could engage Ag after they have entered the medullary sinus, at which point they could continue into the efferent lymphatics and subsequently into downstream LN. LEC that reside in the subcapsule also express high level PD-L1 and may induce deletion of FH T cells activated in an upstream LN.

Our results also provide some insight into the LN microenvironmental influences that contribute to the LN-LEC tolerogenic phenotype. PD-L1 expression is low on the small number of subcapsular LEC that are present in early neonatal life, and increases with the infiltration of lymphocytes and the development of medullary and cortical structures. Nonetheless, expression of normal levels of PD-L1 takes weeks, suggesting a multilayered developmental control involving both molecular and spatial cues. In keeping with this, blockade of LtβR signaling and removal of B cells diminish the representation of PD-L1^hi^ LN-LEC in the medulla, the MFI of PD-L1 on these cells, and the expression of MAdCAM-1 on LEC in the subcapsule. However, removal of T cells is associated with an increase in representation of both PD-L1^hi^ and MAdCAM-1^+^ LN-LEC. LtβR signaling via Ltα_1_β_2_ on DC also controls the expression of MAdCAM-1 on HEV in LN [39], [40], and Ltα_1_β_2_ on B cells is necessary for the production of type I IFNs by subcapsular sinus macrophages [43]. B lymphocytes also promote lymphangiogenesis under inflammatory conditions through their production of VEGF-A [44]–[46]. While LEC in the subcapsule also expressed lower levels of LtβR than those in the cortex and medulla, consistent with ligand induced receptor internalization [47], direct deletion of LtβR on LEC had no effect on either PD-L1 or MAdCAM-1 expression. Thus, the influence of LtβR signaling is indirect, involving a second molecular signal and a second cell. One possible model is that Ltα_1_β_2_^+^ B cells engage other cells that are in communication with LEC in the subcapsule and the medulla. It is also not clear whether the influence of LtβR signaling is on the survival of PD-L1^hi^ LEC, in addition to its influence on the level of PD-L1 expression, which is similar to its influence on MAdCAM-1. While we have implicated the lymphotoxin pathway in regulating high level PD-L1 expression on these LEC, the signaling pathway(s) that control high level PD-L1 expression on LEC residing in the subcapsule, and intermediate level expression by all LEC subpopulations remains to be determined.

In sum, our work has established that the tolerogenic characteristics of LEC subpopulations in peripheral tissues and LN vary. LEC in non-lymphoid tissues express low levels of PD-L1 and PTA suggesting a limited ability to act as tolerogenic APC. However, LEC in the LN medullary sinus are strongly tolerogenic. This suggests a model in which tolerance is engendered as T cells attempt to exit the LN. Overall, these data provide a greater understanding of the cellular and molecular control of LEC as tolerogenic APC, the modulation of which may have therapeutic value in the treatment of autoimmunity and cancer.

## Supporting Information

Figure S1 **TNFα does not control PD-L1 expression on LEC.** LNSC were purified by enzymatic digestion of pooled LN from TNFα^−/−^ and B6 (TNFα^+/+^) mice via CD45 magnetic bead separation, and stained with antibodies specific for CD45, gp38, CD31, PD-L1, and ICAM-1. Representative FACS plot gated off CD45^neg^ gp38^+^ CD31^+^ cells. Numbers indicate percentage of gated population out of total LEC. Data are representative of 2 independent experiments.
(TIF)

Figure S2 **DC do not control PD-L1 expression on LEC.** ***a.*** CD11c-DTR mice were injected subcutaneously with PBS (-DT) or diphtheria toxin (+DT) every other day for a week. After 1 week of treatment, CD45^+^ cells were purified by enzymatic digestion of pooled skin draining LN (inguinal, brachial, axillary) from –DT or +DT treated mice via CD45 magnetic bead separation, and stained with antibodies specific for CD3ε and CD11c. *Left panel*, representative FACS plots gated off of CD3ε^neg^ cells. Numbers indicate percentage of the gated population of total CD45^+^ CD3ε^neg^ cells. *Right panel*, DC were quantified from the percentage generated in (a). ***b.*** LNSC from mice treated in (a) were purified and stained with antibodies specific for CD45, gp38, CD31, and PD-L1. Plot is gated on CD45^neg^ gp38^+^ CD31^+^ cells. Data is representative of 3 independent experiments. ***c.*** LNSC were purified by enzymatic digestion of pooled LN from Batf3^−/−^ and Batf3^+/+^ mice. LNSC were stained with antibodies specific for CD45, gp38, CD31, ICAM-1, and PD-L1, and analyzed by flow cytometry. Plots are gated on CD45^neg^ gp38^+^ CD31^+^ cells. Data is representative of 1 experiment. **p<0.01.
(TIF)

Figure S3 **T cells suppress the expression of PD-L1 by cortical LEC, and MAdCAM-1 expression by medullary LEC.** ***a.*** LNSC were purified by enzymatic digestion of pooled LN and CD45 magnetic bead separation from CD3ε^−/−^ or WT mice. LEC absolute number was calculated from cells that were gated as Dapi^neg^, singlets, CD45^neg^, gp38^+^, CD31^+^ cells by flow cytometry. ***b.*** LNSC were stained with antibodies specific for CD45, gp38, CD31, PD-L1, ICAM-1, MAdCAM-1, and LtβR, and analyzed by flow cytometry. *Left panel*, plots display data using the same gating strategy as in Figure 5. *Right panel*, summary of 3 independent experiments. ***c.*** PD-L1 MFI of PD-L1^hi^ ICAM-1^hi^ and PD-L1^int^ ICAM-1^int^ subpopulations gated on CD45^neg^ gp38^+^ CD31^+^ LN cells of the indicated mice. Data from 3 independent paired experiments. ***d.*** *Left panel*, frozen axillary LN sections of indicated mice were stained with antibodies specific for Lyve-1, and PD-L1. *Right panel*, summary plot of PD-L1 MFI gated on Lyve-1^+^ pixels in the indicated LN location. S = subcapsule C = cortex, M = medulla. Scale bar = 200 µm. ***e.*** *Left panel*, frozen axillary LN sections from indicated mice were stained with antibodies specific for Lyve-1 and MAdCAM-1. *Right panel*, summary plot of MAdCAM-1 MFI gated on Lyve-1^+^ pixels in the LN subcapsule. Scale bar = 200 µm. Staining is representative of multiple fields from 3 independent experiments consisting of 2 separate LN from 3 mice. *p<0.05, ns =  not significant.
(TIF)
