# Supplementary figures and images for: Tolerogenic Properties of Lymphatic Endothelial Cells Are Controlled by the Lymph Node Microenvironment
Source: PLoS One. 2014 Feb 4;9(2):e87740. doi: 10.1371/journal.pone.0087740 (PMC3913631; doi:10.1371/journal.pone.0087740)

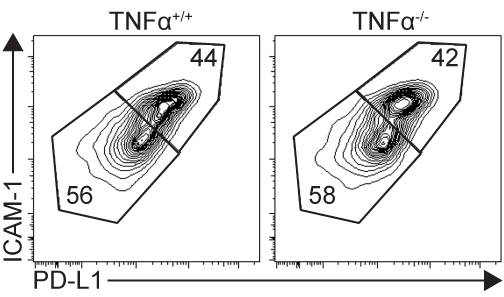

Supplement: Figure S1 — TNFα does not control PD-L1 expression on LEC. LNSC were purified by enzymatic digestion of pooled LN from TNFα−/− and B6 (TNFα+/+) mice via CD45 magnetic bead separation, and stained with antibodies specific for CD45, gp38, CD31, PD-L1, and ICAM-1. Representative FACS plot gated off CD45neg gp38+ CD31+ cells. Numbers indicate percentage of gated population out of total LEC. Data are representative of 2 independent experiments. (TIF) [file pone.0087740.s001.tif]

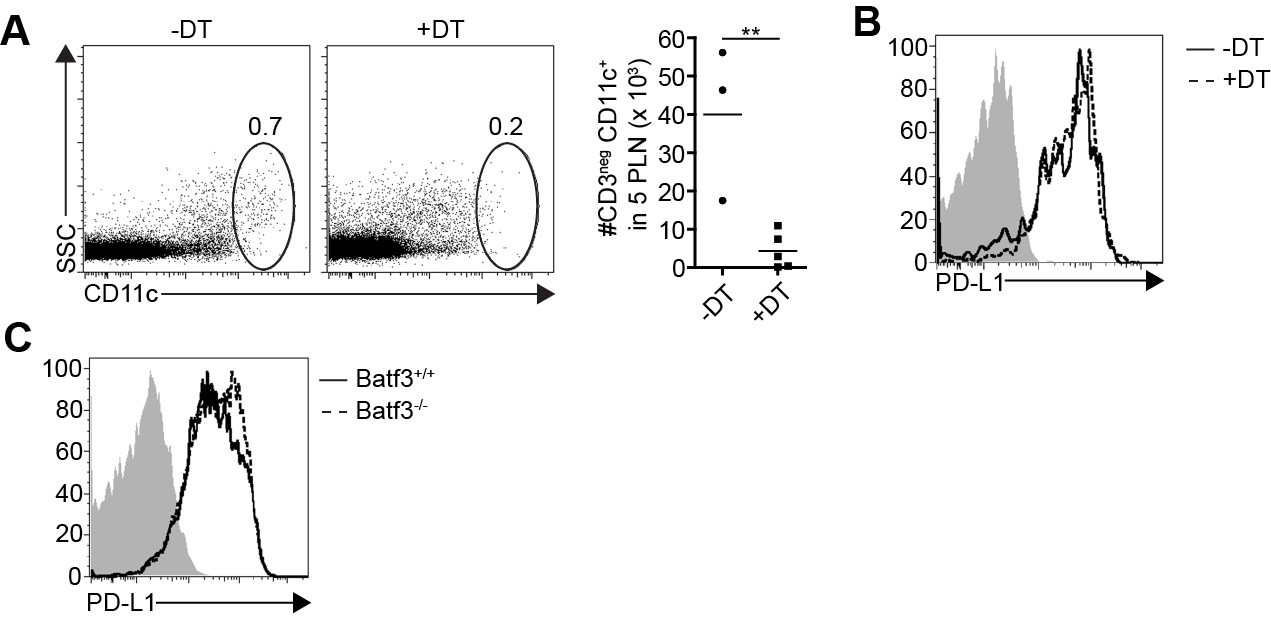

Supplement: Figure S2 — DC do not control PD-L1 expression on LEC. a. CD11c-DTR mice were injected subcutaneously with PBS (-DT) or diphtheria toxin (+DT) every other day for a week. After 1 week of treatment, CD45+ cells were purified by enzymatic digestion of pooled skin draining LN (inguinal, brachial, axillary) from –DT or +DT treated mice via CD45 magnetic bead separation, and stained with antibodies specific for CD3ε and CD11c. Left panel, representative FACS plots gated off of CD3εneg cells. Numbers indicate percentage of the gated population of total CD45+ CD3εneg cells. Right panel, DC were quantified from the percentage generated in (a). b. LNSC from mice treated in (a) were purified and stained with antibodies specific for CD45, gp38, CD31, and PD-L1. Plot is gated on CD45neg gp38+ CD31+ cells. Data is representative of 3 independent experiments. c. LNSC were purified by enzymatic digestion of pooled LN from Batf3−/− and Batf3+/+ mice. LNSC were stained with antibodies specific for CD45, gp38, CD31, ICAM-1, and PD-L1, and analyzed by flow cytometry. Plots are gated on CD45neg gp38+ CD31+ cells. Data is representative of 1 experiment. **p<0.01. (TIF) [file pone.0087740.s002.tif]

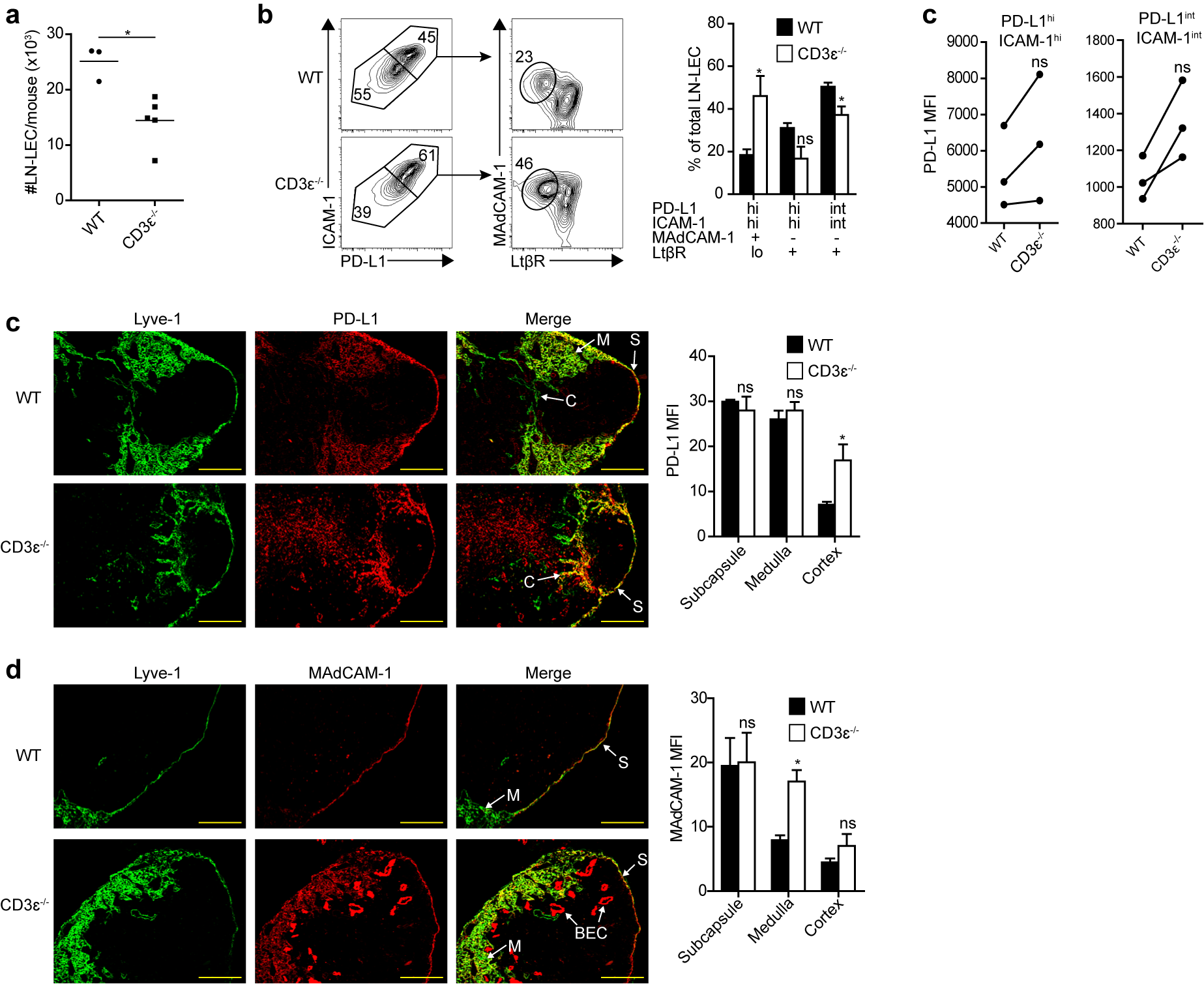

Supplement: Figure S3 — T cells suppress the expression of PD-L1 by cortical LEC, and MAdCAM-1 expression by medullary LEC. a. LNSC were purified by enzymatic digestion of pooled LN and CD45 magnetic bead separation from CD3ε−/− or WT mice. LEC absolute number was calculated from cells that were gated as Dapineg, singlets, CD45neg, gp38+, CD31+ cells by flow cytometry. b. LNSC were stained with antibodies specific for CD45, gp38, CD31, PD-L1, ICAM-1, MAdCAM-1, and LtβR, and analyzed by flow cytometry. Left panel, plots display data using the same gating strategy as in Figure 5. Right panel, summary of 3 independent experiments. c. PD-L1 MFI of PD-L1hi ICAM-1hi and PD-L1int ICAM-1int subpopulations gated on CD45neg gp38+ CD31+ LN cells of the indicated mice. Data from 3 independent paired experiments. d. Left panel, frozen axillary LN sections of indicated mice were stained with antibodies specific for Lyve-1, and PD-L1. Right panel, summary plot of PD-L1 MFI gated on Lyve-1+ pixels in the indicated LN location. S = subcapsule C = cortex, M = medulla. Scale bar = 200 µm. e. Left panel, frozen axillary LN sections from indicated mice were stained with antibodies specific for Lyve-1 and MAdCAM-1. Right panel, summary plot of MAdCAM-1 MFI gated on Lyve-1+ pixels in the LN subcapsule. Scale bar = 200 µm. Staining is representative of multiple fields from 3 independent experiments consisting of 2 separate LN from 3 mice. *p<0.05, ns = not significant. (TIF) [file pone.0087740.s003.tif]
